# Osteolytic lesions as a presenting sign of acute myeloid leukemia: a case report

**DOI:** 10.3389/fonc.2024.1364266

**Published:** 2024-05-01

**Authors:** Jingqian Zhang, Shidai Mu, Li Cai, Lisha Ai, Yaohui Wu

**Affiliations:** ^1^ Institute of Hematology, Union Hospital, Tongji Medical College, Huazhong University of Science and Technology, Wuhan, China; ^2^ Key Lab of Molecular Biological Targeted Therapies of the Ministry of Education, Union Hospital, Tongji Medical College, Huazhong University of Science and Technology, Wuhan, China

**Keywords:** acute myeloid leukemia, osteolytic lesions, myeloid sarcoma, pancytopenia, diagnosis, case report

## Abstract

Osteolytic lesions are infrequently observed in adult patients with acute myeloid leukemia (AML). This report details the case of a 66-year-old male patient who presented with myeloid sarcoma (MS), osteolytic lesion and pancytopenia. Effective treatments were delayed due to diagnostic challenges and the rapid progression of the disease. It is essential to consider AML in the differential diagnosis when faced with a patient presenting osteolytic lesions and pancytopenia.

## 1 Introduction

Acute myeloid leukemia (AML) is a clonal, malignant disease of hematopoietic tissues characterized by accumulation of leukemic blast cells primarily in the marrow. It results in impaired production of normal blood cells, which leads to pancytopenia in blood routine test. AML always manifests with common symptoms such as pallor, fatigue, weakness, fever, bleeding (1).

Myeloid sarcoma(MS), also known as granulocytic sarcoma(GS), extramedullary myeloid tumor and chloroma, is defined by World Health Organization(WHO) as extramedullary tumor masses of myeloid blasts with tissue architecture effaced (2). MS can occur at any sites in the body, with the most common locations being the skin, lymph nodes, mediastinum, testis, intestine, bone and central nervous system(CNS) (3, 4). The incidence of MS in AML varies in different studies, ranging approximately from 0.8% to 10.4% (5–8).

Osteolytic lesions are a common feature in multiple myeloma (MM) and metastatic cancers, such as prostate cancer, thyroid cancer, lung cancer and others. However, osteolytic lesions are rarely reported in adult with AML (9–17).

This report details the case of a patient finally diagnosed with AML who presented primarily with MS, osteolytic lesion and pancytopenia.

## 2 Case report

**Figure 1:**
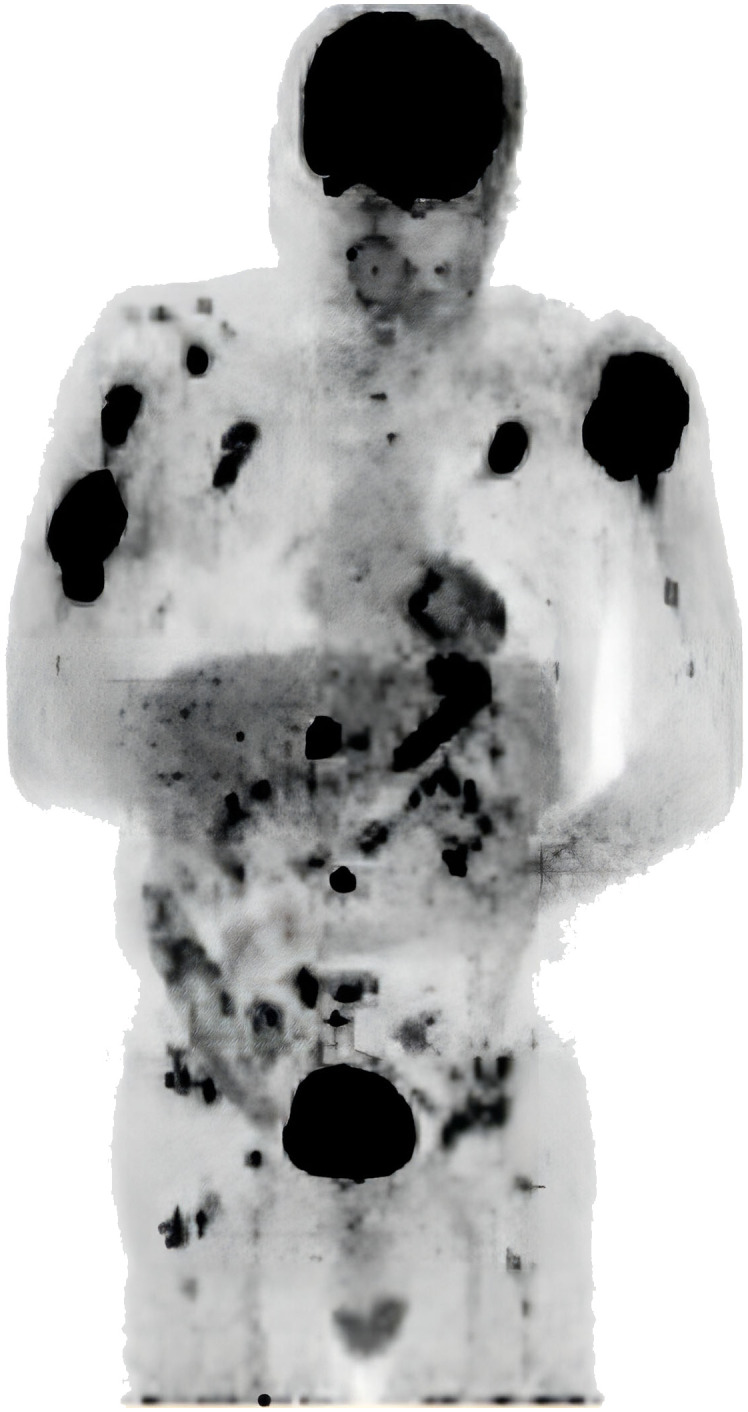
FDG PET/CT showing multiple osteolytic lesions, some lesions accompanied with the formation of soft tissue masses.

A 66-year-old male patient was admitted to the orthopedic department due to a lump on his left upper arm persisting for 2 months, accompanied by pressure pain and pathological fractures occurring 10 days prior. An FDG PET/CT (^18^F-fluorodeoxyglucose Positron Emission Tomography/Computed Tomography) scan revealed multiple osteolytic lesions, observed in the left mandible, right 7^th^ costal rib, left 2^nd^ anterior rib, right clavicle, right scapula, T12, L3, L5, S1 vertebral levels, bilateral proximal humeri, bilateral iliac bones, and femoral neck. Some lesions were accompanied with the formation of soft tissue masses with abnormal concentration of radioactive uptake (**Figure 1**), raising suspicion of metastatic malignancies. The physical examination indicated stable vital signs. The patient had a history of diabetes, managed with metformin, and untreated coronary heart disease. He had no history of previously diagnosed hematological neoplasm.

On the 2^nd^ day of admission, Complete Blood Count (CBC) revealed a hemoglobin level of 78 g/L ↓ (130.0-175.0), erythrocyte count of 2.41 T/L↓(4.3-5.8), total leukocyte count of 3.23 G/L↓(3.5-9.5), and a platelet count of 35 G/L↓ (125–350). The patient’s inflammatory markers showed a high-sensitivity C-reactive protein (hs-CRP) level of 120.81mg/L↑ (<4) and an erythrocyte sedimentation rate (ESR) of 102mm/h↑ (0–15). The patient’s coagulation function, as well as liver and kidney functions, were within normal limits. A puncture of the left humerus was performed on the same day. On the 7^th^ day, the patient developed a high fever up to 39°C, so that the antibiotic treatment had been upgraded.

The puncture of the left humeral bone reported on the 10th day revealed that the principal substance within the bone trabeculae appeared as homogeneous, silty necrotic debris, with faint cellular remnants noted in specific regions. Immunohistochemical staining of cellular remnants was positive for LCA (Leukocyte Common Antigen) and negative for PCK (Cytokeratin) and S-100, indicating neoplastic necrosis with a high probability of lymphopoietic involvement. To elucidate the pathological nature of the tumor further, an incisional biopsy of the left humeral bone was conducted on the 13^th^ day. Immunohistochemical staining of the biopsy specimen reported on the 20^th^ day suggested myeloid sarcoma, with an immunophenotype showing co-expression of monocytic and megakaryocytic markers (**Table 1**).

**Table 1:** Immunohistochemical staining of the left humeral bone.

	Immunohistochemical Markers
Expressed	LCA(+), CD43(+), ERG(partial+), CD34(partial weak+), CD117(partial+), CD68(+), CD33(+), CD13(+), CD163(partial+), CD123(partial+), CD61(partial+), CD38(partial weak+), CD30(partial+), EMA(partial weak+), INI-1 and BRG1(+, no loss), Ki67(LI:about 25%)
Non-Expressed	MPO (–), Lysozyme (–), CD235 (–), CD3 (–), CD5 (–), CD2 (–), CD7 (–), CD20 (–), PAX5 (–), CD79a (–), CD19 (–), MUM1 (–), TdT (–), CD56 (–), PCK (–), CK8/18 (–), SOX10 (–), Desmin (–),SS18-SSX (–), ALK (–), SATB2 (–)

LCA, Leukocyte Common Antigen; CD, Cluster of Differentiation; ERG, ETS Related Gene; EMA, Epithelial Membrane Antigen; INI-1, Integrase Interactor 1; BRG1, Brahma-Related Gene 1; MPO, myeloperoxidase; PAX5, Paired Box 5; MUM1, Multiple Myeloma Oncogene 1; PCK, Cytokeratin; SOX10, SRY (sex-determining region Y)-box 10; ALK, Anaplastic Lymphoma Kinase; SATB2, Special AT-rich Binding Protein 2.

Due to the suspicion of hematological malignancies, the difficulty in controlling the fever and the persistent decrease in platelet count, the patient was transferred to the hematology department on the 17^th^ day of admission. Immediate bone marrow aspiration and biopsy were conducted. Considering the patient’s age of 66, along with pancytopenia and multiple osteolytic lesions, multiple myeloma (MM) was the primary suspicion at that time. During the process of confirming the diagnosis and providing targeted treatment, the patient’s condition continued to deteriorate.

On the 19^th^ day, the patient was transferred to the Hematology Care Unit (HCU) for the clouded consciousness, hypoxia and persistent high fever, prompting suspicion of hemophagocytic lymphohistiocytosis (HLH). The diagnostic criteria were met with a temperature exceeding 38.5°C for over 7 days, ferritin at a dilution of 1:20 measuring 15582.6 μg/L (21.8-275), and an sCD25 level of 10047.17 U/ml (39.26-265.5). However, since the remaining five criteria were either unknown or not satisfied, the diagnosis of HLH remained uncertain.

**Figure 2:**
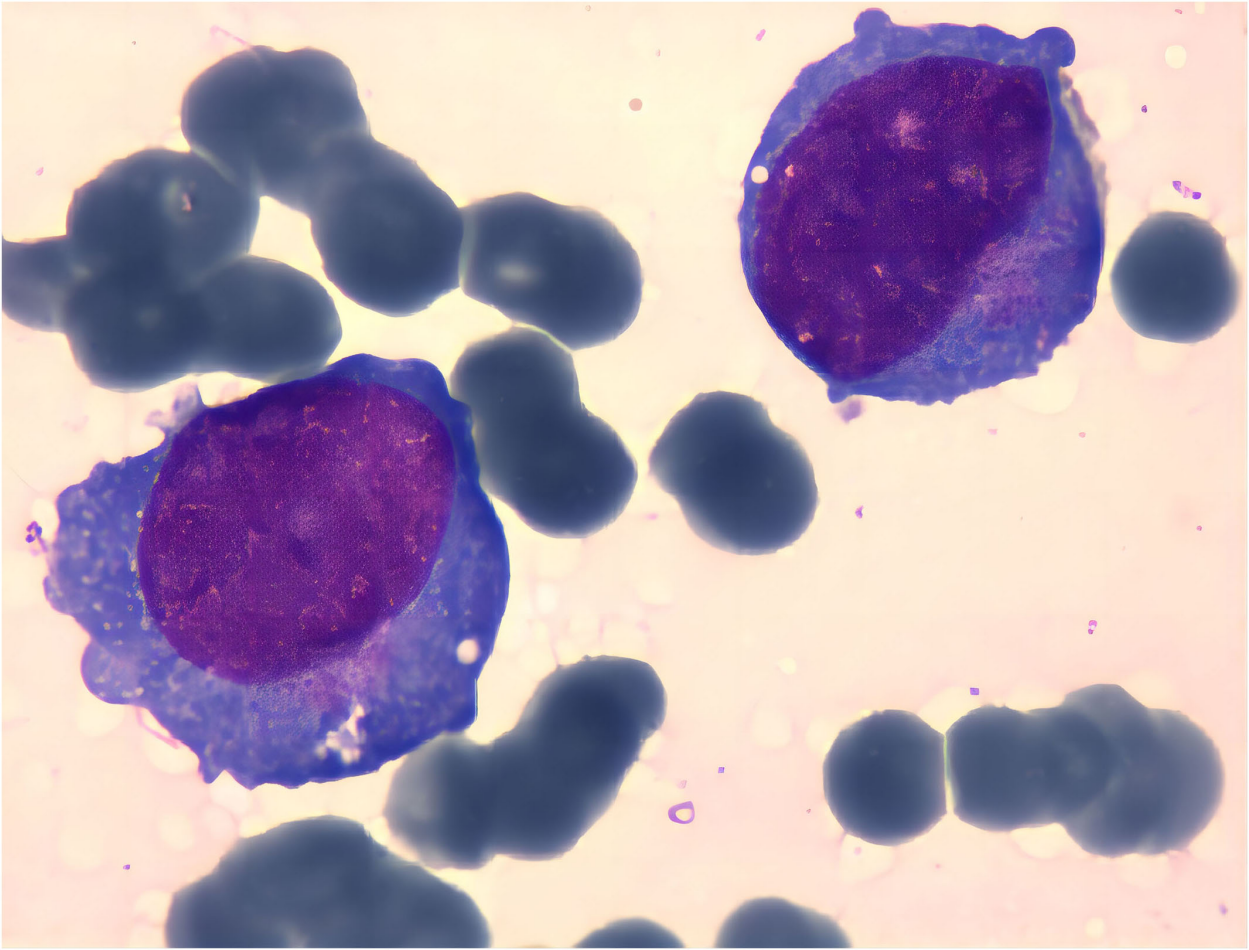
Cell bodies vary in size, with some notably larger. Cytoplasmic content ranges from scant to abundant, appearing grayish-blue upon staining. Some cells exhibit cytoplasmic extensions, containing fine pinkish granules. Nuclei display both regular and irregular shapes, with uniformly fine chromatin. Variability in nucleolar prominence is observed.

**Figure 3:**
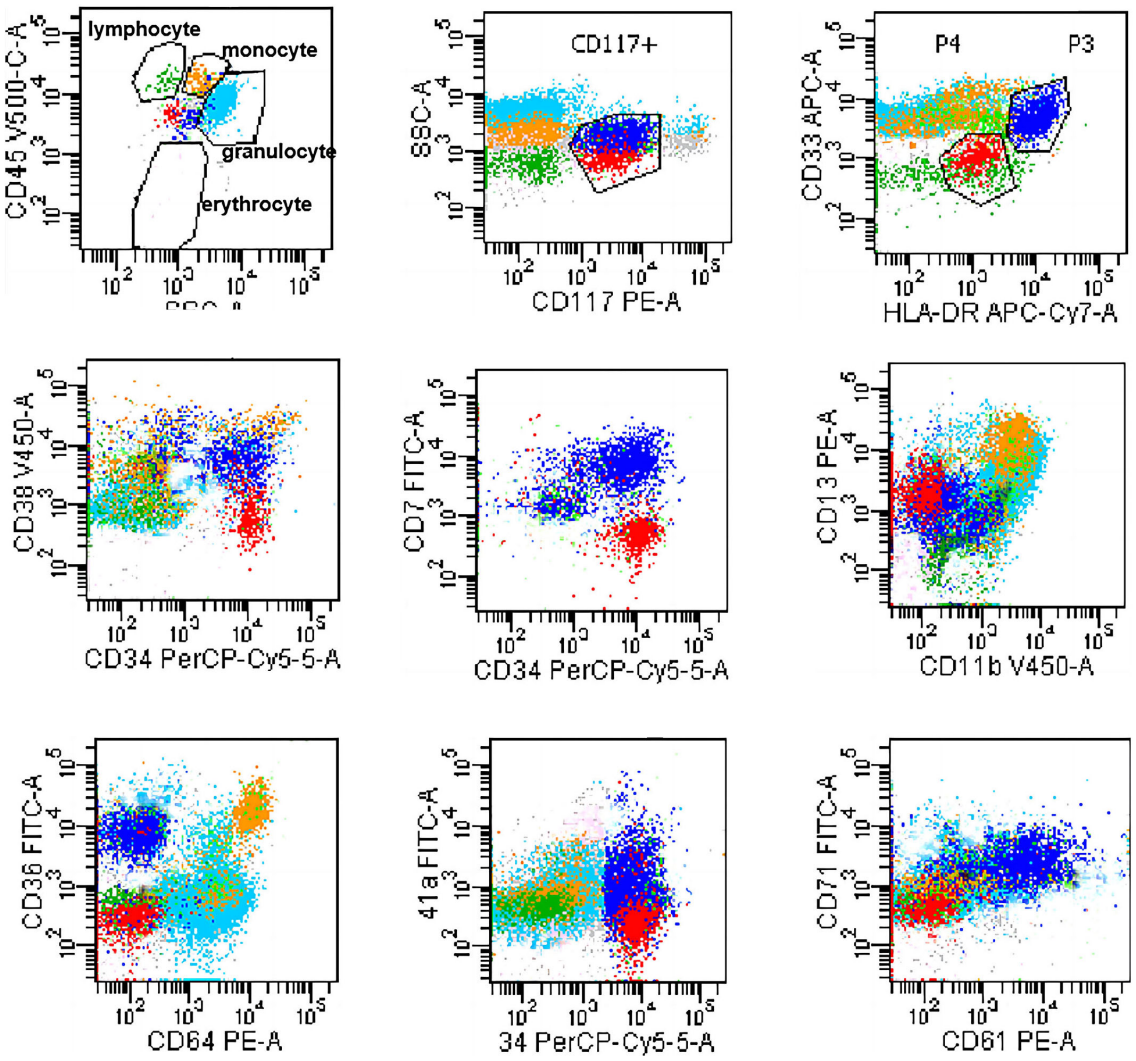
Flowcytometry dot plot showing two populations of phenotypically aberrant myeloid progenitor cells. Blue dots: 6.95% Blasts; Red dots: 2.64% Blasts.

Serum electrophoresis reported on the 20^th^ day revealed no evidence of monoclonal gammopathy. Immunohistochemical staining of bone marrow biopsy reported on the 20^th^ day indicated features consistent with AML (**Table 2**). Bone marrow aspiration reported on the 21^st^ day indicated that primitive blood cells accounted for 29% (**Figure 2**). Cytochemical staining revealed positivity for POX(Peroxidase), partial positivity for ANAE (Alpha-Naphthyl Acetate Esterase), and negativity for CE (Chloroacetate Esterase) and PAS(Periodic Acid-Schiff), indicative of myeloid differentiation. The peripheral blood smear showed 33% primitive blood cells. Immunophenotyping reported on the 23^rd^ day revealed two populations of phenotypically aberrant myeloid progenitor cells within the nucleated cells (**Table 3**, **Figure 3**). MM-FISH (fluorescence *in situ* hybridization) reported on the 24^th^ day revealed multiple abnormalities, including amplification of chromosome 1q21 (copy number = 3), 1p32, 4p16, 11q13, 14p32 and 17. Karyotype analysis showed an absence of the mitotic phase. The above results supported the diagnosis of AML.

**Table 2:** Immunohistochemical staining of bone marrow biopsy.

	Immunohistochemical Markers
Expressed	CD34(20%+), CD117(15%+), MPO(+), Ki67(+), CD61(minimal+), CD68(sporadic+), CD138(1%+), CD163(sporadic+)
Non-Expressed	TDT (–), CD3 (–), CD20 (–), CD56 (–), CD235a (–), EMA (–), PCK (–)

CD, cluster of differentiation; TDT, Terminal Deoxynucleotidyl Transferase; MPO, myeloperoxidase; EMA, Epithelial Membrane Antigen; PCK, Cytokeratin.

**Table 3:** Immunophenotyping on the bone marrow aspiration.

	6.95% Blasts	2.64% Blasts
Expressed Immunophenotypic Markers	CD34, CD117, CD13, CD38, CD33, HLA-DR st, CD7, CD36, CD61, CD71, CD41a	CD34, CD117, CD13, HLA-DR dim
Non-Expressed Immunophenotypic Markers	CD19, MPO, cCD3, cCD79a, CD11b, CD16, CD22, CD10, CD4, CD15, CD64, CD14, CD56, CD123, CD11c, CD42b	CD7, CD19, CD38, MPO, cCD3, cCD79a, CD11b, CD16, CD22, CD10, CD4, CD15, CD33, CD36, CD64, CD14, CD56, CD123, CD61, CD11c, CD71, CD41a, CD42b
Interpretation	considerable likelihood of primitive megakaryocytic cells.	phenotypically aberrant myeloid progenitor cells

Unfortunately, on the 21^th^ day of admission, the patient suffered respiratory and cardiac arrest. Despite rescue efforts being ineffective, the patient was discharged at the request of the family. The exact diagnose was not confirmed until the day of discharge.

## 3 Discussion

This report details the case of a 66-year-old man presenting with a lump on his left upper arm and PET/CT findings indicating multiple osteolytic lesions. The diagnosis was obscured and delayed due to atypical bone symptoms. The obscured diagnosis and rapid disease progression resulted in inadequate treatment, significantly impacting the patient’s outcome.

The diagnosis of MM was ruled out based on bone marrow aspiration and biopsy results, along with a negative serum electrophoresis for monoclonal gammopathy. The diagnosis of AML was established through morphological and immunological evidence in the bone marrow, and the presence of myeloid sarcoma (MS). AML manifesting as MS and multiple osteolytic lesions causes significant diagnostic dilemma.

As previously reported, due to the lack of adequate immunohistochemical test, MS has often been misdiagnosed as lymphoproliferative disorder, particularly diffuse large B-cell lymphoma (DLBCL), Hodgkin lymphoma, small lymphocytic leukemia, and T-cell lymphoma. Additional common misdiagnoses encompass myeloma, thymoma, and extramedullary hematopoiesis (18). In our case, the immunohistochemical finding from the left humerus puncture with limited markers suggested neoplastic necrosis and a high likelihood of lymphopoietic neoplastic necrosis. The subsequent immunohistochemical test of incisional biopsy, which included sufficient molecular markers, confirmed the diagnosis of MS. This underscores the importance of adequate immunophenotyping in confirming MS.

Osteolytic lesions are not a common feature of AML. The multiple osteolytic lesions are typically associated with metastatic cancers and MM. There have been only a few reported cases of AML presenting with osteolytic lesions in patients older than 14 years (**Table 4**). The osteolytic lesions were considered as a sarcomatous manifestation (19) or tumor-mediated bone erosion (13). A biopsy of an osteolytic lesion in an AML M7 patient revealed consistency with an extramedullary myeloid cell tumor with megakaryocyte differentiation (12). However, the specific pathophysiological mechanism of bone lytic lesions in AML remains elusive. Osteolytic lesions were considered as a poor prognosis in AML (19) or an advanced disease state at the course of AML transforming from MDS (11). Previous cases also reported relief of bone pain through local irradiation (9, 11, 19). Unfortunately, effective treatment options were limited by the rapid progress of the disease in this case.

**Table 4:** Patients (≥15 year-old) with AML presenting with osteolytic lesion.

Author(Published year)	Age-Sex	Main manifestations	AML subtype (FAB)
Dharmasena F (9) (1986)	27M	Pain and swelling of his left shoulder	AML M7(suspected previously undiagnosed CGL)
Marsh, W. L (10) (1986)	26F	Sporadic dull sternal tenderness → severe lower back pain, hip pain, and a rigid abdomen	AML M4
Daniele Be (11) (1996)	68F	left hip pain	Transformation into AML from CMML
Lima, C. S (19) (2000).	17M	weakness, fever, severe lumbar pain and weight loss.	AML
Muler, J. H (12) (2002).	32M	back pain in his lumbar area	AML M7
Krauss, K (13) (2004).	58M	developed lower back pain radiating to the right posterior thigh	AML M1
Seifis (14) (2010)	21F	lower back pain, anorexia, weight loss and fever	AML M2
Geetha, N (20) (2015).	17M	Pain in right shoulder and right chest wall, and intermittent fever.	AML M6
Chambers, Isaac (15) (2016)	62F	increasing weakness, confusion, and a fall.	Transformation into AML from PV
Su, Z (16) (2017).	49M	Chest pain	AML
Duval, Guillaume (17) (2019)	82M	A decline in general health and pain in right shoulder	AML transformation of myeloproliferative syndrome

M, male; F, female; AML, acute myeloid leukemia; CGL, chronic granulocytic leukemia; CMML, chronic myelomonocytic leukemia; PV, polycythemia vera.

A peripheral blood smear was conducted performed concurrently with bone marrow aspiration in this case. The accessibility and efficiency of peripheral smear morphology render it a valuable routine diagnostic measure, especially in cases of pancytopenia. When conducted during the initial hospitalization, it can accelerate diagnosis and enable timely interventions.

Due to the patient’s passing and the discrepancy between the initial and definitive diagnoses, as well as the absence of the mitotic phase in cytogenetics, molecular analysis and AML-NGS data are not accessible. Nevertheless, molecular or cytogenetic studies would have been highly beneficial in similar cases to determine if a specific cytogenetic or molecular profile confers a higher risk for the manifestation of multiple osteolytic lesions or MS.

## 4 Conclusion

This case implies that AML must be considered in the differential diagnosis in face of a patient presenting with bone lesions and pancytopenia. Conducting a simple peripheral blood smear, ensuring adequate immunophenotyping of tissue sections, and performing timely bone marrow aspiration are crucial steps to ensure a correct diagnosis and prevent delays in initiating effective treatment.

## Data availability statement

The original contributions presented in the study are included in the article. Further inquiries can be directed to the corresponding author. Requests to access these datasets should be directed to ailisha@hust.edu.cn.

## Ethics statement

The studies involving humans were approved by Institutional Review Board of Tongji Medical College, Huazhong University of Science and Technology. The studies were conducted in accordance with the local legislation and institutional requirements. Written informed consent for participation was not required from the participants or the participants’ legal guardians/next of kin in accordance with the national legislation and institutional requirements. Written informed consent was obtained from the individual(s) for the publication of any potentially identifiable images or data included in this article.

## Author contributions

JZ: Writing – original draft, Writing – review & editing. SM: Writing – original draft, Writing – review & editing. LC: Resources, Writing – review & editing. LA: Funding acquisition, Writing – review & editing. YW: Funding acquisition, Writing – review & editing.
